# Spindle associated membrane protein 1 (Samp1) is required for the differentiation of muscle cells

**DOI:** 10.1038/s41598-017-16746-y

**Published:** 2017-11-30

**Authors:** Mohammed Hakim Jafferali, Ricardo A. Figueroa, Mehedi Hasan, Einar Hallberg

**Affiliations:** Department of Neurochemistry, Stockholm University, Svante Arrhenius väg 16B, SE-106 91 Stockholm, Sweden

## Abstract

Muscles are developed and regenerated in a differentiation process called myogenesis, which involves components of the nuclear envelope. We have investigated Samp1 (Spindle Associated Membrane Protein 1), a transmembrane nuclear envelope protein, which interacts with emerin and lamin A, both of which are linked to Emery-Dreifuss muscular dystrophy (EDMD). We found that the levels of Samp1 increased seven-fold during differentiation of mouse C2C12 muscle progenitor cells. To test if Samp1 could have a role in myogenesis we developed stable C2C12 knockdown cell lines expressing short hairpin RNA targeting Samp1 expression. The Samp1 depleted C2C12 cells displayed normal mobility and normal distribution of emerin and lamin A. However, Samp1 depletion increased ERK signaling and completely blocked differentiation of C2C12 cells, which failed to express myogenic marker proteins and failed to form myotubes. The block in myogenesis in Samp1 depleted cells was completely rescued by ectopic expression of RNAi resistant human Samp1, showing that Samp1 is required for muscle differentiation.

## Introduction

Emery-Dreifuss muscular dystrophy (EDMD) is linked to genes encoding proteins located in the nuclear envelope (NE)^1–3^. The NE^4–6^ surrounds the nucleus and consists of two concentric lipid membranes, the nuclear lamina, the nuclear pores and LINC (Linker of Nucleoskeleton and Cytoskeleton) complexes, which span the NE and connects the cytoskeleton with the nuclear lamina^7^. The inner nuclear membrane of the NE displays a far higher degree of protein complexity than previously anticipated^8–10^. Most of the several hundreds of the NE proteins identified today, display a remarkable diverse tissue specificity with only 17% of NE proteins shared between muscle, liver and leukocytes^11^. Paradoxically, laminopathies displaying tissue specific pathologies are linked to genes encoding widely expressed NE proteins, which apparently manifest their dysfunction in yet undefined tissue specific cellular processes. EDMD patient cells display centrosome detachment from the nucleus, a phenotype that can be evoked by experimental silencing of emerin, lamin A, nesprin-1, nesprin-2 and Samp1^12–14^. This suggests that in muscle cells, these proteins cooperate in a common LINC complex mediated mechanism, which becomes disrupted in EDMD.

The assumption that NE proteins whose expression are increased in a certain tissue also have important tissue specific roles has laid the ground for several investigations^15,16^. NE proteins that are highly expressed in muscle have for example been subjected to several studies focused on investigating their potential role in muscle development and effects on myogenesis have been reported from RNAi experiments^17,18^. For example, knockdown of lamin A, emerin and Net25 each reduced myogenesis due to hyperactivation of Erk signaling^19,20^, which counteracts the necessary cell cycle exit step in muscle cell differentiation. A few other NE proteins have been reported to affect myogenesis by repositioning muscle specific genes in the nucleus and thereby affecting their expression^18^. Combined silencing of Net39, Tmem38a and WFS1 gave a stronger repression of myogenesis than individual silencing of these proteins, suggesting that some NE proteins may have a concerted mechanism of action^18^. How muscle development and regeneration of new muscle cells contribute to muscular dystrophy diseases^21^ needs to be further investigated.

Here we show that the expression of Samp1 (Spindle Associated Membrane Protein 1)^12^ increased several-fold during myogenesis. Samp1 is an INM protein which binds directly to emerin^22,23^ and interacts with lamin A^24^, both of which are linked to EDMD. We further investigated the process of differentiation of cycling mouse C2C12 myoblasts into myotubes and show that differentiation of myoblasts was completely abolished in Samp1 depleted cells, an effect that could be rescued by ectopic expression of human Samp1. The strong and clear effect of Samp1 expression in promoting C2C12 differentiation suggests a central and important role for Samp1 in myogenesis.

## Results

### Samp1 expression is induced during myogenic differentiation

**Figure 1:**
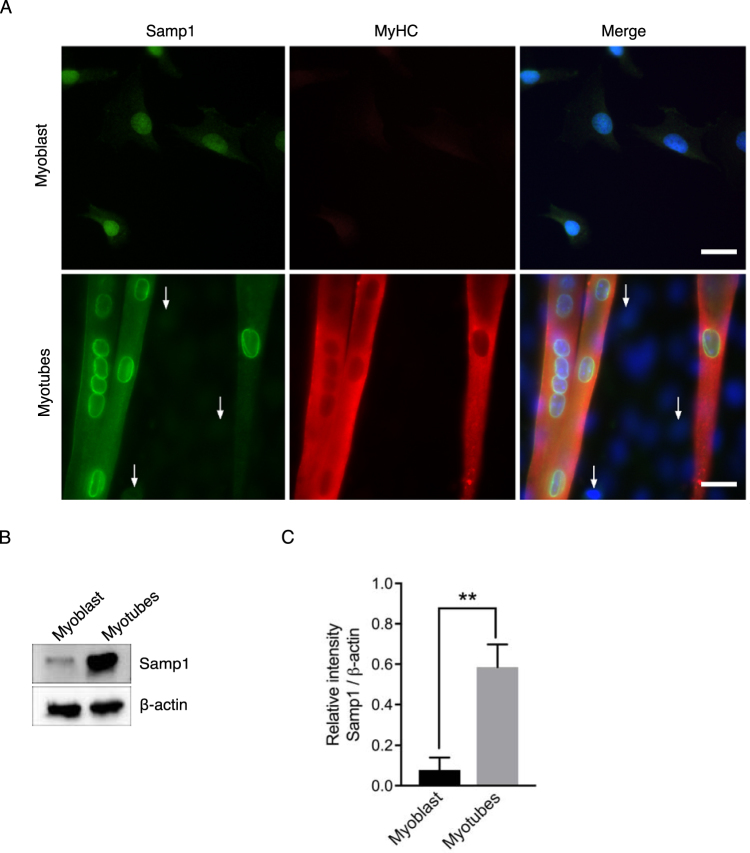
Samp1 expression increases during myoblast differentiation. (**A**) Immunofluorescence microscopy analysis of Samp1 (green) and myosin heavy chain (MyHC) (red) in C2C12 myoblasts and myotubes. Draq5 (blue) was used to stain for DNA. Note the intense anti-Samp1 staining in the rims of nuclei inside myotubes, but not in the isolated myoblasts (arrows). Scale bar, 20 μm. (**B**) Whole cell extracts of mouse C2C12 myoblasts and myotubes were analyzed by Western blotting using anti-Samp1 antibodies. β-actin was used as loading control. (**C**) Quantification showing a seven-fold increase in Samp1 levels in myoblast compared with myotubes, two tailed Students t-test (P = 0,0024), mean ± SD, n = 3.

Tissue expression surveys using proteomics^16^ and immunohistochemistry^25^ show that Samp1 expression varies widely between different tissues and cell types, but is relatively high in skeletal and heart muscle. This prompted us to study expression of Samp1 during muscle differentiation. Using a well characterized mouse skeletal C2C12 cell *in vitro* model, we investigated the expression of Samp1 during myogenic differentiation. C2C12 cells proliferate as undifferentiated cycling myoblasts when cultured in growth medium containing 20% fetal bovine serum (referred to as proliferation media, PM). Differentiation was induced by withdrawal of fetal bovine serum and addition of 2% horse serum to the medium (referred to as differentiation medium, DM) for 6 days. Myogenesis was evident by C2C12 myoblasts transforming into multinucleated myotubes that stained positive for myosin heavy chain (MyHC) (Fig. 1A), a marker for myogenic differentiation. In undifferentiated C2C12 cells Samp1 immunostaining was hardly detectable compared to secondary controls (Fig. 1A). In contrast, the nuclei inside myotubes displayed intense rim staining (Fig. 1A). Whole cell lysates of myoblast or myotube cultures were harvested and analyzed by Western blot using antibodies specific for Samp1 (Fig. 1B) and using β-actin as loading control (see also Fig. S1). Undifferentiated C2C12 myoblasts displayed only a weak signal, whereas differentiated C2C12 myotubes gave a strong signal. Quantification of the Western blot signals showed a significant 7-fold increase of the Samp1 protein level in differentiated myotubes as compared to undifferentiated myoblasts (Fig. 1C). Similar results were obtained using lamin B1 as loading control (Fig. S1B). The results show that Samp1 expression is dramatically increased during myogenic differentiation.

**Figure 2:**
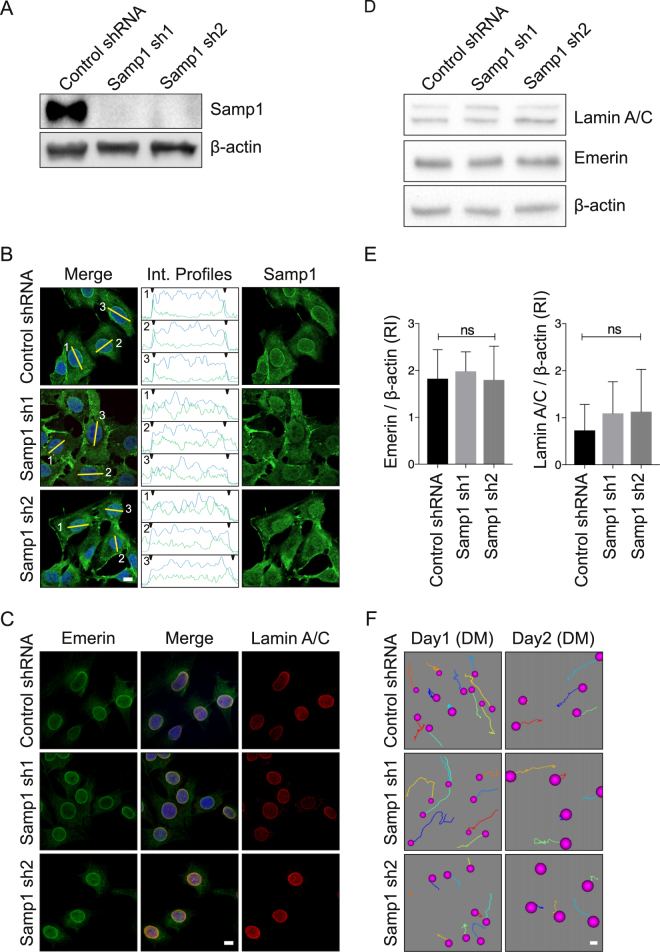
Establishment of Samp1 knockdown cell lines. (**A**) Whole cell extracts derived from separate mouse C2C12 cell lines stably expressing either of two Samp1-targeting shRNAs (Samp1 sh1 and Samp1 sh2) and a non-targeting hairpin sequence (Control shRNA) and grown under non-differentiation conditions, were analyzed by Western blotting using anti-Samp1 antibodies. Note the complete knockdown of Samp1 expression in the sh1 and sh2 KD cells, respectively. β-actin was used as loading control. (**B**) Stable shRNA cell lines were grown on glass bottom dish, fixed and stained for Samp1 (green) and Draq5 (blue) and imaged using confocal microscopy. Pixel intensity profiles (along yellow lines as indicated) for Samp1 (green) and Draq5 (blue) are shown for 3 cells in each category. Scale bar, 10 µm. Note the absence of intensity peaks (denoted by arrow heads) at the nuclear rims of Samp1 KD cell lines. (**C**) Confocal Immunofluorescence microscopy of shRNA cell lines stained using antibodies specific for Emerin (green) and Lamin A/C (red) shows no difference between KD Samp1 cell lines and control cells. Scale bar, 10 µm. (**D**) Representative Western blot of whole cell lysates of shRNA cell lines using antibodies specific for Emerin and Lamin A/C. (**E**) Quantification of Western blotting for Emerin and Lamin A/C in the shRNA cell lines. (**F**) Time-lapse microscopy. The control shRNA and the stable shRNA cell lines were grown to confluence in PM and continuous phase contrast images were captured for 6 hrs on Day 1 and 3 hrs on Day 2 in DM, respectively. Scale bar, 10 µm. The movement of individual cells were tracked and represented by different colored traces. The positions of nuclei in the first image in the series are indicated with magenta colored dots.

Next, we investigated whether Samp1 could play a role in myogenic differentiation. To test this, we generated stable knockdown cell lines by infecting proliferating myoblasts with lentivirus like particle suspensions carrying control shRNA or Samp1 specific shRNAs (Samp1 sh1 and Samp1 sh2). In the Samp1 knockdown (KD) cells Samp1 was not detectable by Western blot analysis (Fig. 2A) nor by immunofluorescence (IF) microscopy analysis (Fig. 2B), suggesting that Samp1 was completely depleted in both KD cell lines. Furthermore, there was no effect on the distribution or levels of lamin A/C (Fig. 2C–E), emerin (Fig. 2C–E) or centrosome position (Supplementary Fig. S2B) in the KD C2C12 myoblasts. In previous studies short term (96 h) siRNA mediated silencing of Samp1 resulted in emerin mislocation^22^ and centrosome detachment^12^ in HeLa cells. These discrepancies are most likely due to cell type specific differences because the NE protein repertoire shows a high degree of diversity between different tissues^11^. In support of this idea, short term shRNA knockdown of Samp1 expression in C2C12 cells did not affect distribution of emerin (Fig. S2C) nor cause centrosome detachment (Fig. S2A). NIH 3T3 fibroblasts depletion of Samp1 resulted in inhibition of cell migration in response to wound healing^24^. In the present study, time-lapse microscopy showed that migration of the Samp1 KD cells was somewhat slower compared control cells (Fig. 2F). But both the KD Samp1 cell lines and the control shRNA cell lines remained in motion throughout the entire period (Supplementary movie S1 and S2).

### Samp1 is required for myogenesis

**Figure 3:**
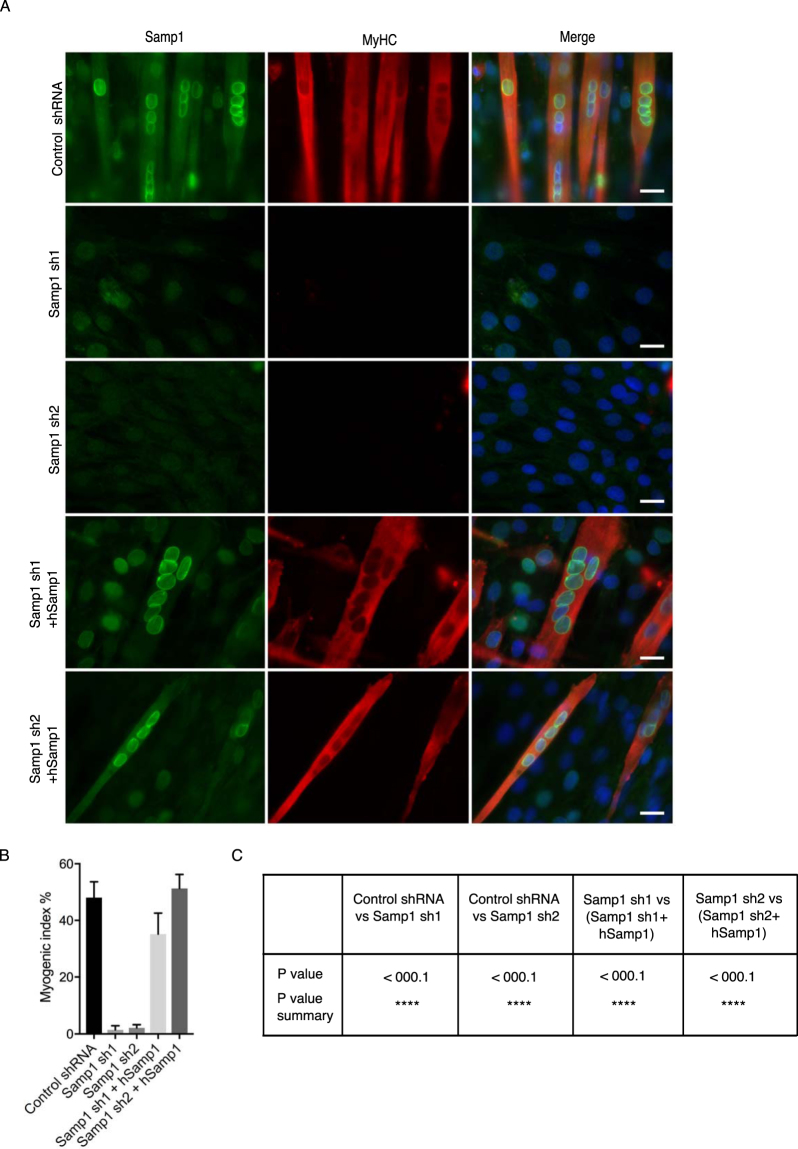
Samp1 is required for myogenesis. Stable control sh, KD Samp1 cell lines and KD Samp1 cell lines transduced with cDNA encoding the short isoform of human Samp1 were cultured for 6 days in DM. (**A**) The cells were then fixed and immunostained using antibodies specific for Samp1 (green) and MyHC (red). Draq5 (blue) was used to stain for DNA. Scale bar, 20 μm. Note that neither of the two Samp1 knockdown cell lines were able to form myotubes and that this effect was completely rescued by ectopic expression of hSamp1. (**B**) Quantification of myogenic differentiation expressed as myogenic index, which is the percentage of nuclei in MyHC positive cells. Error bars indicate SEM. (**C**) Statistical analysis of data in B, two tailed Students t-test (n = 3).

Next, we investigated whether Samp1 could play a role in myogenesis. The KD Samp1 sh cell lines were cultured in DM and analyzed by IF microscopy using antibodies specific for Samp1 and MyHC. After six days the shRNA control cells had fused to form myotubes expressing both MyHC and Samp1 (Fig. 3A) as expected. In contrast, almost no myotubes were formed in the KD Samp1 shRNA cell cultures grown in DM. The myogenic index dropped from 50 to only a few % in Samp1 depleted cells (Fig. 3B and C). Interestingly, the myogenesis block in the Samp1 sh1 and Samp1 sh2 cell lines was completely rescued by ectopic expression of the short isoform of human Samp1 (Samp1a, according to the nomenclature used in^24^), which is resistant to the mouse shRNA (Fig. 3). This strong and clear result shows that Samp1 is required for myogenesis and that the short isoform is sufficient to restore myogenesis. In comparison to previous studies^18^ using combinatorial KD of selected muscle specific NETs, the effect of KD of Samp1 alone was much stronger, indicating a key role for Samp1 in differentiation of C2C12 cells.

### Depletion of Samp1 during differentiation inhibits expression of myogenic transcription factors and activates MAP kinase signaling

**Figure 4:**
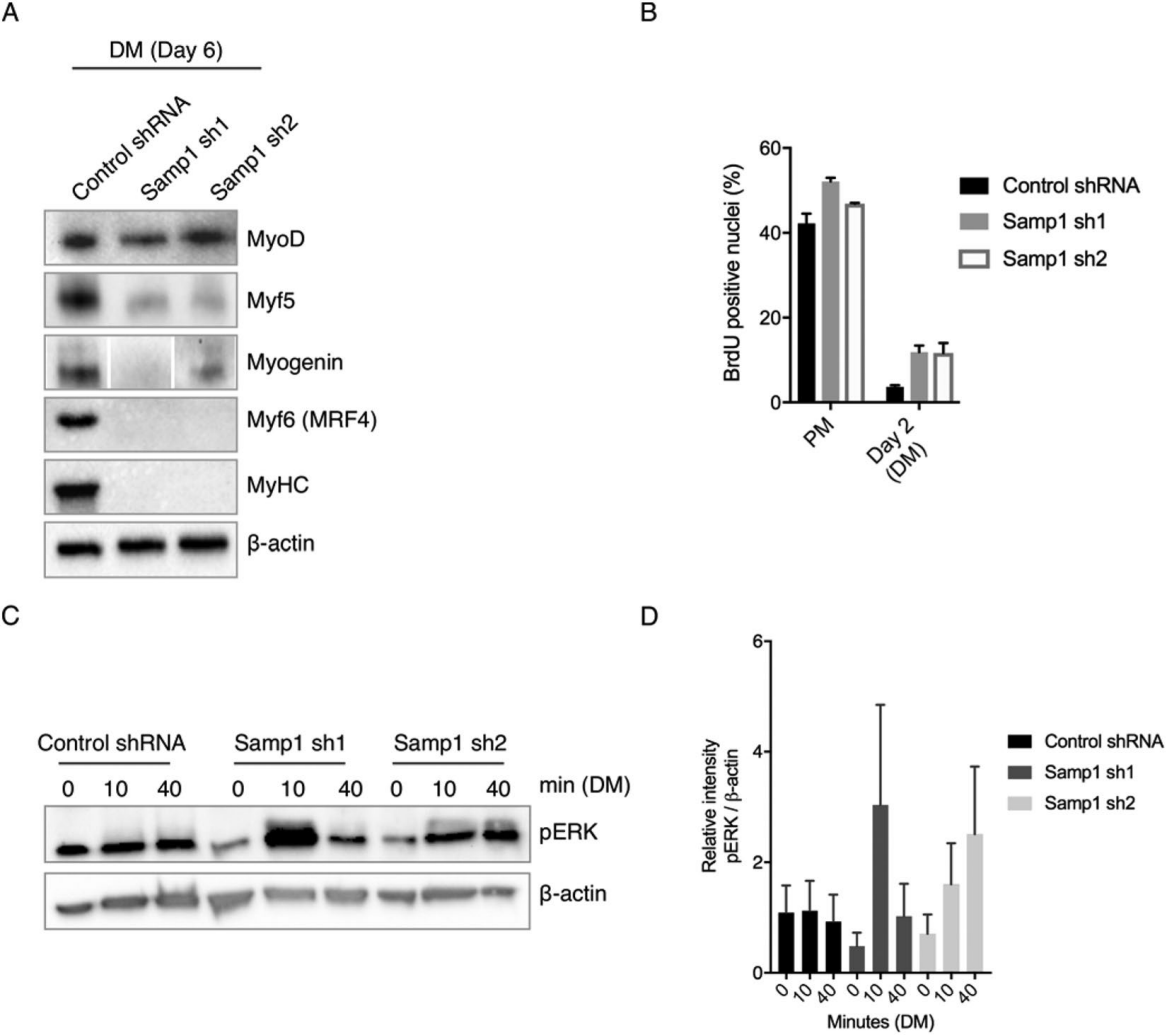
Effect on expression of myogenic markers, cell proliferation and MAP kinase signaling in Samp1 knockdown cell lines. Stable Samp1 depleted sh cell lines were allowed to differentiate for 6 days in DM media. (**A**) Whole cell extracts were subjected to Western blot analysis using antibodies specific for MyoD, Myf5, Myogenin, Myf6 (MRF 4) and MyHC in the KD cell lines. β-actin was used as loading control. Note the dramatic decrease in expression of all markers except MyoD in Samp1 depleted cells. (**B**) Quantification (% ± S.D.) of BrdU-positive nuclei in control and Samp1-depleted C2C12 cultures in PM and on day 2 after shift to DM. The data comes from two experiments. At least 200 (PM) or 1000 (DM) nuclei were counted per condition. (**C** and **D**) Stable shRNA cell lines were allowed to reach confluence in PM and then shifted to DM. After 0, 10 and 40 min incubation the whole cell extracts were collected and subjected to Western blot analysis using antibodies specific for MAP kinase, pERK (**C**). β-actin was used as loading control. (**D**) Quantification (% ± S.D.) of Western blot analysis described in **C** (two independent experiments). Note the increase in pERK signaling after 10 min (Samp1 sh1) and 40 min (Samp1 sh2) shift to DM in Samp1 depleted cell lines.

During myoblasts differentiation three sequential steps occurs^26^. First, expression of muscle specific transcription factors and cell cycle exit takes place. Second, migration of myoblasts. Third, attachment and fusion of cells to form myotubes. To investigate the expression of myogenic transcription factors, stable KD Samp1 cell lines and control cells were allowed to differentiate for six days in DM. Whole cell lysates were collected and the expression of four key myogenic transcription factor and the myogenic structural differentiation marker myosin heavy chain (MyHC) were analyzed by Western blotting. Remarkably, Samp1 depleted cell lines did not show detectable levels of Myf6 (MRF4) or MyHC, whereas myogenin and Myf5 were expressed at reduced levels (Fig. 4A).

Cell cycle exit is an important step in cell differentiation. In order to determine the effect of Samp1 depletion on cell proliferation, we performed BrdU labeling of C2C12 cells grown in PM and 2 days after initiation of differentiation in DM. Although BrdU incorporation decreased in all samples on day 2, the Samp1 depleted cells displayed a higher incorporation of BrdU compared to control cells (Fig. 4B), indicating that cell cycle exit was inhibited. Failure to exit the cell cycle was also observed after depletion of lamin A, emerin, Man1 or NET25 as a result of hyperactivation of the mitogen activated protein kinase (MAPK) signaling^19,20^. A branch of the MAPK signaling cascade, the extracellular signal-regulated kinase (ERK), has been shown to be abnormally activated in cardiomyopathy associated with EDMD caused by mutations in the LMNA and EMD genes^27,28^. Therefore, we investigated if Samp1 depletion could have an effect on ERK activation. Indeed, we found increased pERK levels after 10 min (Samp1 sh1) and 40 min (Samp1 sh2) exposure to DM (Fig. 4C). The mechanism behind ERK activation as a response to misregulated expression of certain NE proteins remain elusive. To summarize, we can conclude that the complete inhibition of myogenesis in Samp1 depleted cells cannot be explained by dislocation of emerin or lamin A/C (c.f., Fig. 2C–E)^19,20^. In contrast, Samp1 appears to have an important role in myogenesis independently of emerin or lamin A/C.

## Discussion

Biochemical and genetic variations in the components of the NE causes a broad range of clinical phenotypes, including muscular dystrophies, cardiomyopathies, neuropathies and lipodystrophies. Skeletal muscles are susceptible to abnormalities of the nuclear envelope, with mutations in lamin A giving rise to both EDMD and LGMD^29,30^. Two thirds of EDMD cases are linked to emerin or lamin A^31^, both of which are interaction partners of Samp1^23,24^. Samp1 also interacts with the LINC complex protein, Sun1^22,23^, which is also implicated in EDMD. A recent study^32^ showed that Samp1 distributed abnormally, in some, but not all EDMD patient cells. The strong and clear requirement for Samp1 in differentiation of C2C12 myoblasts presented here may provide new insights in the pathological development in EDMD. Samp1 is an interesting novel candidate disease gene for the wide variety of EDMD cases not associated by mutations in LMNA or EMD. A subset NE proteins, including emerin, LEMD2/Net25, Net39, Tmem38A, WFS1 and Samp1, are upregulated in muscles and considered candidates for promoting myogenesis^15,16,18^.

In this study, switching to DM evoked a quick and transient increase in ERK signaling in Samp1 depleted cells. Similar responses have been observed in cells with reduced expression of lamin A, emerin or Net25/LEMD2^19,20^. The myogenic pathway is controlled by the myogenic regulatory factors (MRF’s), MyoD, Myf5, myogenin and MRF4 (Myf6). Expression of these MRF proteins are spatially and temporally controlled^33^. MyoD and Myf5 were considered to be the main determination factor of myogenesis but a study by Kassar-Duchossoy *et al*.^34^ has revised the epistatic relationship of MRFs, so that both Myf5 and MRF4 act upstream of MyoD in determining the myogenic lineage^34^ in embryogenesis. In adult myogeneis, MyoD maintains differentiation potential of skeletal myoblasts and Myf5 regulates the proliferation rate and homeostasis. The other MRF’s myogenin and MRF4 are not required for cell development and maintenance but, expression of myogenin is necessary for formation of myotubes and myofibers^35^. The inhibition of the myogenic pathway in cells depleted of Samp1 seen here and of other NE proteins^19,20^ may be tied to ERK signaling, which is known to contribute to the JAK1-STAT1-STAT3 pathway that promotes proliferation and prevents premature myoblast differentiation^36,37^. In accordance, the levels of Myf5 compared to controls were lower (6 days after switching to DM) in cells depleted of Samp1 (Fig. 4A) and (4 days after switching to DM) in cells with reduced expression of Net25/LEMD2, emerin or Man1^20^. How these different NE proteins can have similar effects on ERK signaling can only be a matter for speculation at this stage, but it suggests that they may all interplay with a common yet unidentified cellular function. The NE is known to interact with chromatin and have impact on gene expression. Net39, Tmem38A and WFS1 repositioned myospecific genes to the nuclear periphery to facilitate their repression^18^. However, the effect on ERK signaling seen in cells depleted of Samp1 (Fig. 4C–D), lamin A, emerin in Net25/LemD2^19,20^ is apparently too quick to depend on the transcription/translation machinery. Instead, one could speculate if the distribution and/or turnover of MAPKKs or MAPKKKs or phosphatases that act on ERK could be affected by altered expression of certain NE proteins. In fact, several signaling events are known to take place at the INM and the NE is known to sequester signaling molecules^38^.

Interestingly, in a parallel, but independent, study of iPSCs, Samp1 expression induced a rapid differentiation of iPSCs despite culturing under pluripotency conditions^39^. Together these two studies support the idea that Samp1 can have a general differentiation promoting activity and may be involved in differentiation of a variety of cell types. The mechanism behind this is not understood at present. The lamin A dependent (“A-tether”) tethering of chromatin to the nuclear periphery plays an important role in differentiation^40^. A selection of INM proteins, including Man1, LemD2/Net25, emerin and Samp1 was recently tested as candidate for chromatin binding mediators of the A-tether with negative results^25^. In our study, no effect was observed on lamin A expression in Samp1 depleted cells arguing against an indirect effect on lamin A. Unraveling the mechanistic details behind the role of Samp1 in myogenesis will be an appealing topic for future investigations.

## Materials and Methods

### Plasmid Construction

Two shRNA sequences that were effective in silencing the expression of all murine Samp1 isoforms were designed. These were Samp1 sh1, encoded by 5′-GTGCCTTCTTGTTGTTCACTA-3′, and Samp1 sh2 encoded by 5′-GAGCAGTACAATGGCTTTCAA-3′. Synthetic sense and antisense oligonucleotides were annealed and cloned into linearized pLKO.1-TRC lentiviral vector between the sites AgeI and EcoRI. Positive clones were confirmed by sequencing in both directions. To provide a control, scrambled control shRNA was used. The human Samp1a-YFP plasmid^12^ was cloned into pLJM1-EGFP lentiviral expression vector between the sites NdeI and AgeI, subsequently removing the EGFP between AgeI and EcoRI and adding a linker containing a stop codon. The resulting plasmid expressing human Samp1a is referred to as hSamp1. The pLKO.1-TRC cloning vector was a gift from David Root (Addgene plasmid #10878)^41^. Scrambled shRNA (Addgene plasmid #1864)^42^ and pLJM1-EGFP (Addgene plasmid #19319)^43^ was gifts from David Sabatini.

### Cell culture and transduction

Murine skeletal C2C12 myoblasts (ATCC) were cultured and maintained in proliferation medium (PM): Dulbecco’s modifed Eagle’s medium (DMEM) supplemented with 20% fetal bovine serum and 1% penicillin-streptomycin (v/v) at 37 °C in a humidified atmosphere containing 5% CO_2_. Differentiation was initiated in confluent myoblast cell populations by shifting them to differentiation medium (DM): DMEM supplemented with 2% horse serum and 1% penicillin-streptomycin. Myotube formation was monitored for up to 6 days.

To generate stable Samp1 depleted myoblast cell lines, wild type C2C12 myoblasts were plated at 30% density and transduced with lentivral suspension containing either Samp1 sh1 or Samp1 sh2 shRNAs. For control, C2C12 myoblasts were transduced with scrambled control shRNA lentiviral suspension. 48 h post transduction the cells were selected in proliferation medium supplemented with puromycin, initially at a concentration of 4 μg/ml for 7 days and maintianed at 2 μg/ml.

For the rescue experiments stable Samp1 sh1 or Samp1 sh2 cell lines were cultured in PM at 30% density. The cells were transduced with hSamp1a lentiviral particles. 72 h post transduction upon reaching 80% confluency, cells were shifted to DM. Myotube formation was monitored using phase contrast microscopy for up to 6 days. For all the experiments, Samp1 depleted and control cell lines were cultured in puromycin free PM and DM. For short term shRNA experiments, wild type C2C12 myoblasts were plated at 30% density and transduced with lentivral suspension containing either Samp1 sh1, Samp1 sh2 or control shRNAs and then analysed within 96 hrs.

### Antibodies

Polyclonal rabbit antibodies specific for Samp1 previously described in Buch *et al*.^12^ were used at a dilution of 1:500. Mouse antibodies against anti-phosphoERK (pERK) and rabbit antibodies against Myf5 were kind gifts from I.Faye and J. Nedergaard, respectively. The following antibody concentrations were used: mouse anti-myogenin 1:100 (F5D, DSHB); mouse anti-myosin heavy chain 1:50 (MyHC) (Mf-20, DSHB); mouse anti-MyoD 1:1000 (SC 32758, SCBT); rabbit anti-Myf6 1:1000 (ab 82842, Abcam); mouse anti-β-actin 1:5000 (A 5441, Sigma); mouse anti-Lamin A/C 1:100 (131c3, SCBT); rabbit anti-emerin 1:500 (HPA000609, Atlas antibodies) mouse anti-pericentrin 1:1000 (ab 28144, Abcam); mouse anti-BrdU (Sigma-Aldrich); horseradish-peroxidase-coupled donkey anti-mouse IgG (NA931, GE health care) or donkey anti-rabbit IgG (NA934, GE health care); Alexa Fluor 488 goat anti-rabbit IgG (A11008, Invitrogen); Alexa Fluor 568 goat anti-mouse IgG (A11001, Invitrogen); Alexa Fluor 568 goat anti-rabbit IgG (A11011, Invitrogen).

### Lysate preparation and Western blot analysis

C2C12 myoblast or myotube cultures were rinsed with 1x PBS and scraped using a rubber policeman. The cell pellets were then collected, rinsed with 1x PBS and lysed in 7 M urea and 1% TX-100 with protease inhibitor for 20 min in ice. A small aliquot of the lysate was saved for bicinchoninic acid assay (BCA) for protein concentraion measurement. The remaining cell lysates were mixed with equal volumes of 2X Sample buffer containing 200 mM DTT and boiled for 5 min at 95 °C. Equal volumes of whole cell lysates with equal protein concentrations were loaded on precast 10% SDS-PAGE gels (Bio-Rad, #456–1094). SDS-PAGE separated proteins were transferred onto PVDF membranes (Bio-Rad, #1060002), blocked with 5% dry milk in PBS-T (blocking solution) for 1 h at RT. The membranes were incubated with primary antibodies in the blocking solution for 1 h or overnight at 4 °C. After three 10 min washes in PBS-T, the membranes were incubated with secondary antibodies in blocking solution for 1 h. After four 10 min washes in PBS-T, the membranes were subjected to ECL detection (Amersham ECL prime, GE Healthcare, #rpn2235). The emitted chemiluminescent signal was analyzed by ChemiDoc XRS + imaging system (Bio-Rad).

### BrdU Incorporation

Cells were seeded and allowed to grow for a minimum of one day on glass bottom dishes. Cells were then differentiated or processed prior to reaching confluency for day 0 samples. For BrdU incorporation media were supplemented with 10 µM BrdU from a 10 mM stock in PBS and pulsed for a duration of 3 h after which cells were fixed and processed for immunofluorescence with an added acid post-permeabilization treatment in 1 M HCL for 1 h. At least 1000 cells or 20 random fields of view per sample were manually scored for BrdU incorporation.

### Immunofluorescence

Cells were grown on glass bottom dishes. The cells were washed twice with PBS, fixed with 3.7% PFA in PBS for 20 min on ice and permeabilized with 0.5% TX-100 in PBS for 5 min on ice. After three washes with PBS the cells were blocked in 2% BSA in PBS-T (blocking buffer) for 1 h and incubated with primary antibodies in blocking buffer for 1 h or overnight. After four washes with blocking buffer the samples were incubated with secondary antibodies containing Draq5 (1:5000) in blocking buffer for 1 h. After additional washes with PBS-T, the glass-bottomed dishes were imaged. Draq5 was used to stain the DNA. 1x PBS was used in all steps.

### Imaging and image processing

Imaging was performed on a Leica DM/IRBE 2 epi-fluorescence microscope equipped with a Hamamatsu Orca-ER CCD camera, using a 40 × 1.25 NA oil immersion objective for fluorescence imaging and a 20 × 0.6 NA phase contrast objective for live cell imaging. The system was temperature controlled with a Box and Cube system (Life Imaging Services) for live cell imaging. Micro manager 1.4 was used to control the system and acquire images^44^. Fluorescent images were collected using GFP, N3 and Y5 filter cubes (Chroma Technology Corporation, VT, USA) for Alexa 488, Alexa 568 and Draq5 respectively. Confocal imaging was performed on a Yokogawa CSU22 spinning disk confocal system equipped with an extra filter wheel (Prior), connected to a Zeiss M100 microscope body with a piezo Z scanner (Piezosystems, Jena) and a 63 × 1.4 NA objective, a Hammatsu Flash V2 sCMOS camera and a NordicCombiner (CrystaLaser) solid state laser source with 488 nm, 568 nm and 633 nm laser lines. To control this system and acquire images MicroManager 1.4 was used.

All image quantification and processing was performed using Fiji/ImageJ^45^. Myogenic index was described as the percent nuclei present in cells expressing myosin heavy chain quantified from manually analysing a minimum of 7 images from random fields of view counted using Cell Counter plugin. Mitotic index was quantified by manually counting the percent nuclei positive for BrdU staining using Cell Counter plugin. Intensity profiles over the nuclear rim was generated using a line width of 16 pixels and the profile tool. Profiles wear normalized according to (I-I_1st percentile_)/I_99th percentile_ for each curve. Tracking of cells from time-lapse movies was based on manual segmentation of nuclei from phase contrast images and recorded using the TrackMate plugin^46^. Images of pericentrin from confocal stacks are projections of maximal intensity to visualize pericentrin regardless of focal plane in the confocal stack. Images are linearly adjusted for optimal visibility using the same settings for all images in each panel allowing for direct comparison between the conditions.

### Lentivirus production

Lentivirus like particles (lenti-VLS:s) were produced in HEK293T cells grown in DMEM supplemented with 10% fetal bovine serum and 1% penicillin-streptomycin (v/v). HEK293T cells were transiently transfected in 10-cm plates with murine shRNA (Control shRNA or Samp1 sh1 or Samp1 sh2) or human Samp1a vector and packaging mix (psPAX2 and pMD2.G) at a ratio of 2.5:1:1 using X-tremgene 9 DNA transfection reagent (Roche). Fresh media was replaced 12 h post transfection and lenti-VLP containing medium was collected at 24 and 48 h. The collected medium was sterile filtered and concentrated by centrifugation at 10 000 g 4 °C for 4 h through a 20% sucrose cushion as described in Jiang *et al*.^47^. The collected pellet were re-suspended in PBS overnight, aliquoted and stored at −80 °C until used for transduction. psPAX2 (Addgene plasmid #12260) and pMD2.G (Addgene plasmid #12259) was a gift from Didier Trono.

## Electronic supplementary material

Supplementary Materials

Supplementary Video 1

Supplementary Video 2
