# Publisher Correction: Spindle associated membrane protein 1 (Samp1) is required for the differentiation of muscle cells

**DOI:** 10.1038/s41598-018-22524-1

**Published:** 2018-03-07

**Authors:** Mohammed Hakim Jafferali, Ricardo A. Figueroa, Mehedi Hasan, Einar Hallberg

**Affiliations:** Department of Neurochemistry, Stockholm University, Svante Arrhenius väg 16B, SE-106 91 Stockholm, Sweden

Correction to: *Scientific Reports* 10.1038/s41598-017-16746-y, published online 30 November 2017

An error occurred after publication resulting in an incorrect copyright year being listed in the PDF version of this Article. This error has now been corrected in the PDF version of the Article; the HTML version was correct from the time of publication.

