# Supplementary material for: Spindle associated membrane protein 1 (Samp1) is required for the differentiation of muscle cells
Source: Sci Rep. 2017 Nov 30;7:16655. doi: 10.1038/s41598-017-16746-y (PMC5709512; doi:10.1038/s41598-017-16746-y)
Supplement: Supplementary file 3 — Supplementary Video 2 [file 41598_2017_16746_MOESM3_ESM.pdf]

Spindle associated membrane protein 1 (Samp1) is required for the differentiation of muscle cells.

Mohammed Hakim Jafferali, Ricardo A. Figueroa, Mehedi Hasan, Einar Hallberg*

Department of Neurochemistry, Stockholm University, Sweden
* Correspondence: einar.hallberg@neurochem.su.se

Supplementary figure S1

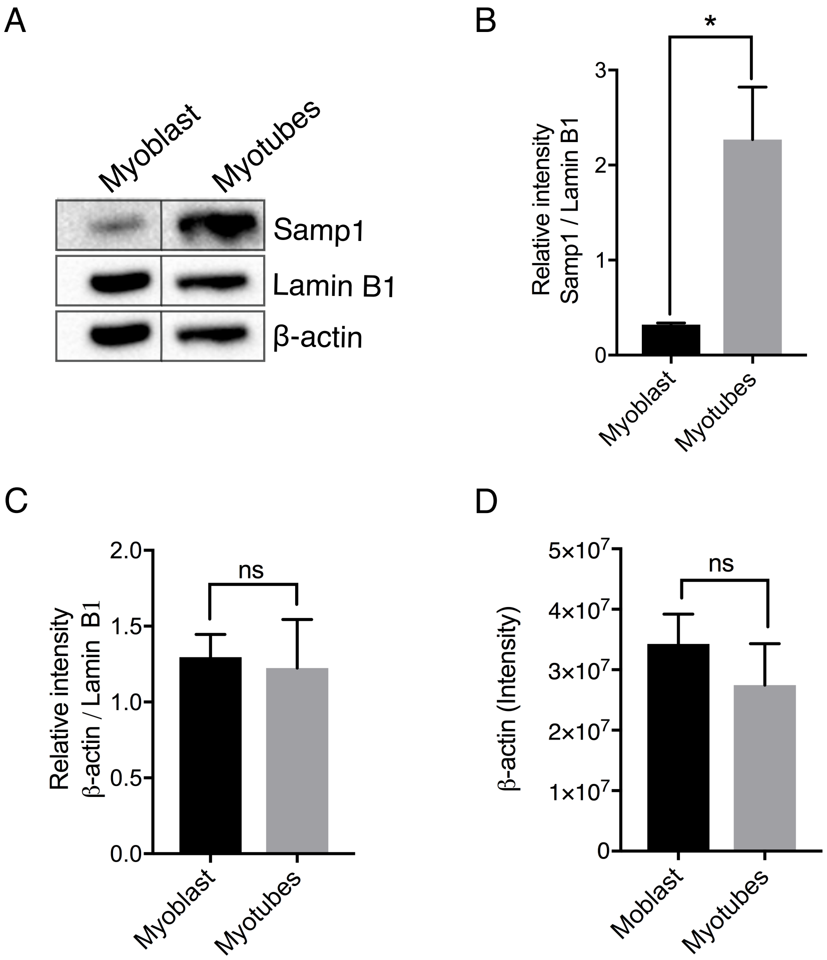

Supplementary Fig S1. Lamin B and β-actin as loading controls in differentiating C2C12 cells. (A) Representative Western blots of Samp1 in myoblasts and myotubes using Lamin B1 and β-actin as loading controls. (B) Quantification (%±S.D., n=3) of Samp1 levels with Western blotting using Lamin B1 as loading control show a significant ($p<0.05$) 7-fold increase in C2C12 myotubes compared to myoblasts. (C) Quantification (the ratio β-actin/lamin B1 ±S.D., n=3) of Western blot analysis of Lamin B1 and β-actin in myoblasts and myotubes, respectively. (D) Quantification of β-actin levels analysed by Western blotting show no significant difference between myotubes and myoblast (n=3).

Supplementary figure S2

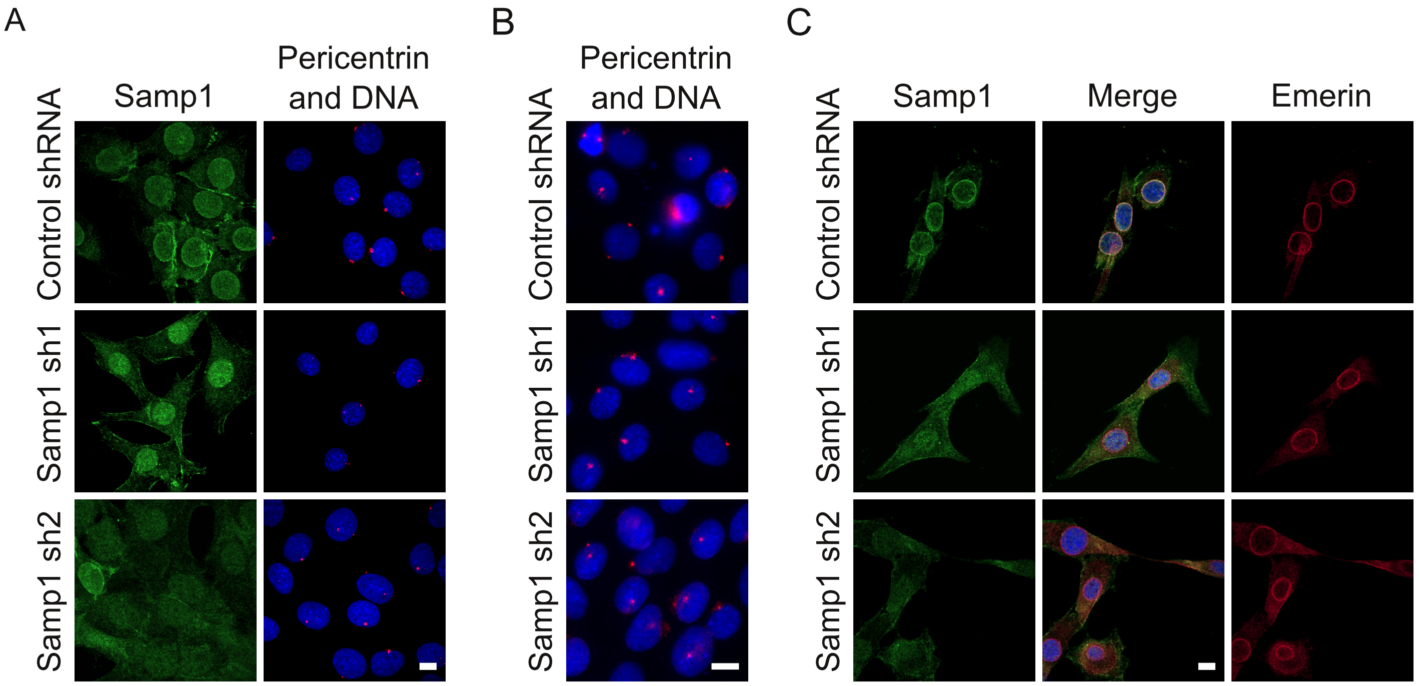

Supplementary Fig S2. **Short term Samp1 knockdown in C2C12 cells**. C2C12 cells were treated with shRNA and incubated for 96 hrs (A and C) and compared to shRNA cell lines (B). (A) Short term shRNA treated C2C12 cells were fixed and stained for Samp1 (green), pericentrin (red) and Draq5 (blue) Projections of confocal Z-stacks are shown. Scale bar, 10 µm. (B) Stable Samp1 depleted and control shRNA cell lines were fixed and stained for pericentrin (red) and Draq5 (blue), analyzed by epifluorescence microscopy. Scale bar, 20 µm. Neither short term shRNA treated C2C12 cells nor shRNA cell lines showed detachment of centrosomes from the nuclear envelope. (C) Short term shRNA treated C2C12 cells fixed and stained for Samp1 (green), Emerin (red) and Draq5 (blue). Confocal equatorial sections show no difference in Emerin distribution. Scale bar, 10 µm.

Supplementary Movie S1. Phase-contrast time-lapse movie of control and Samp1 depleted cell lines on Day 1 in DM. Note that the nucleus on the Samp1 depleted cells migrate as the control cells.

Supplementary Movie S2. Phase-contrast time-lapse movie of control and Samp1 depleted cell lines on Day 2 in DM. Note that the nucleus on the Samp1 depleted cells migrate as the control cells.

Supplementary figure S3

Figure 1B:

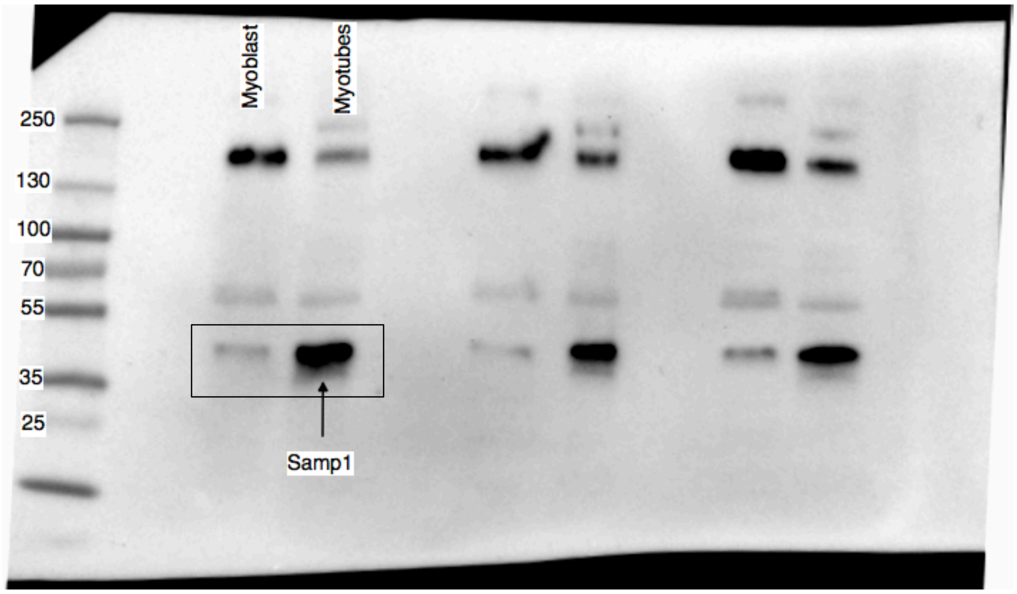

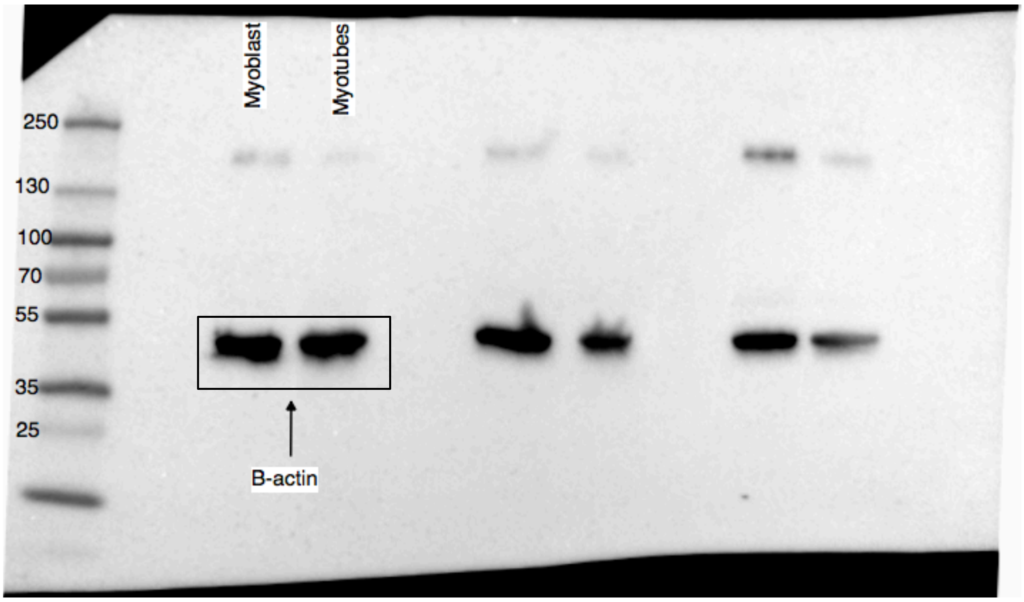

Figure 2A:

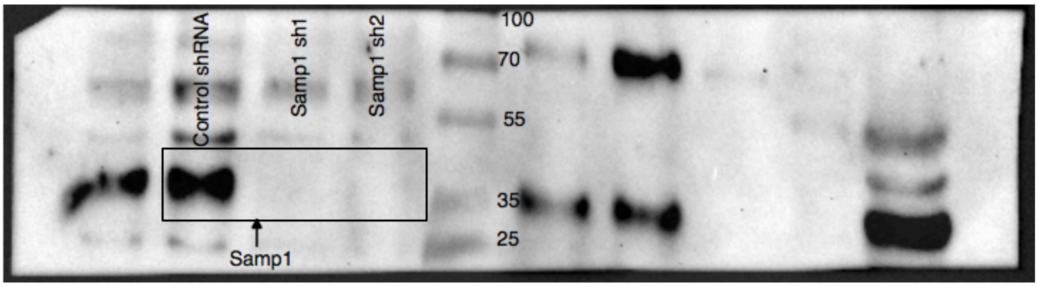
Control shRNA
Samp1 sh1
Samp1 sh2
100
70
55
35
25
Samp1

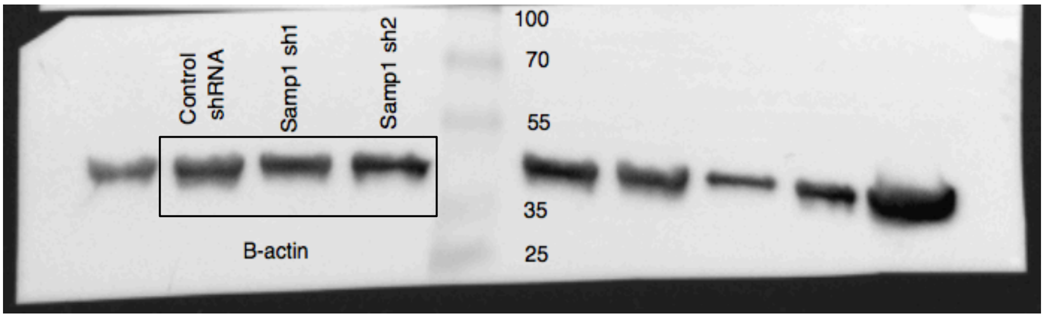
Control shRNA
Samp1 sh1
Samp1 sh2
100
70
55
35
25
B-actin

Figure 2D:

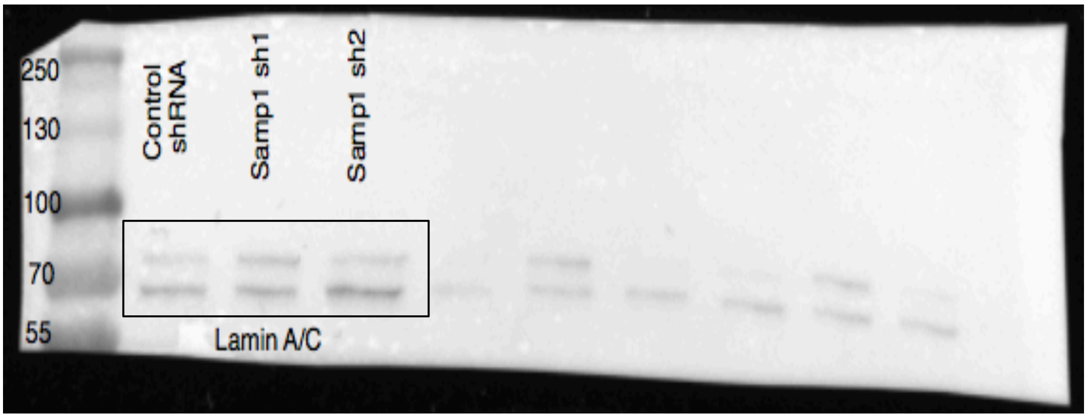

Figure 2D:

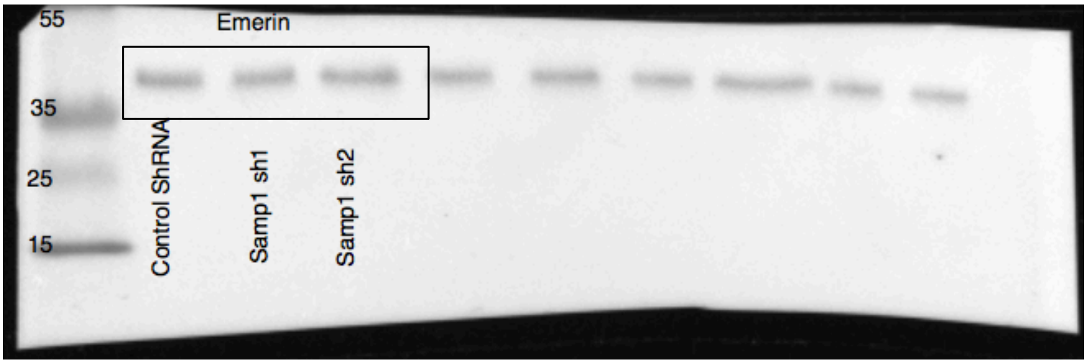

Figure 2D:

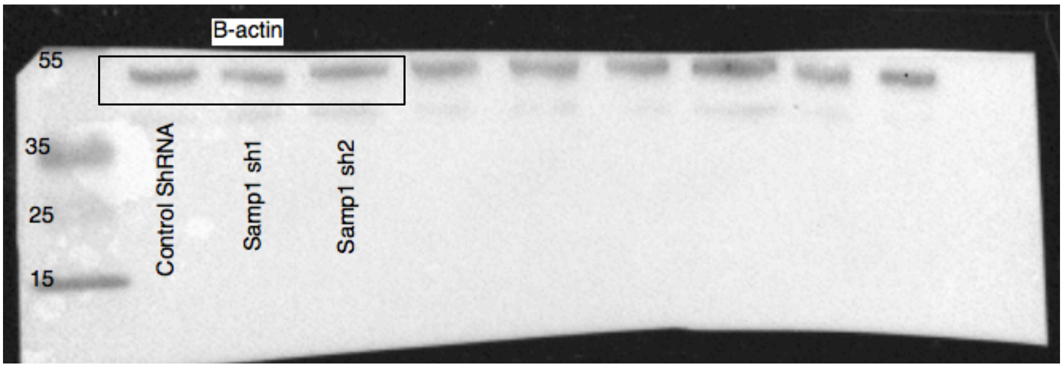

Figure 4A:

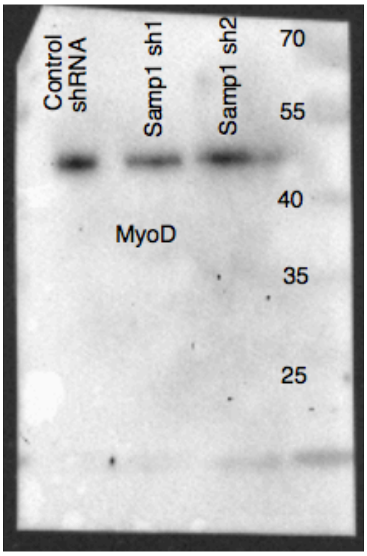

:Figure 4A:

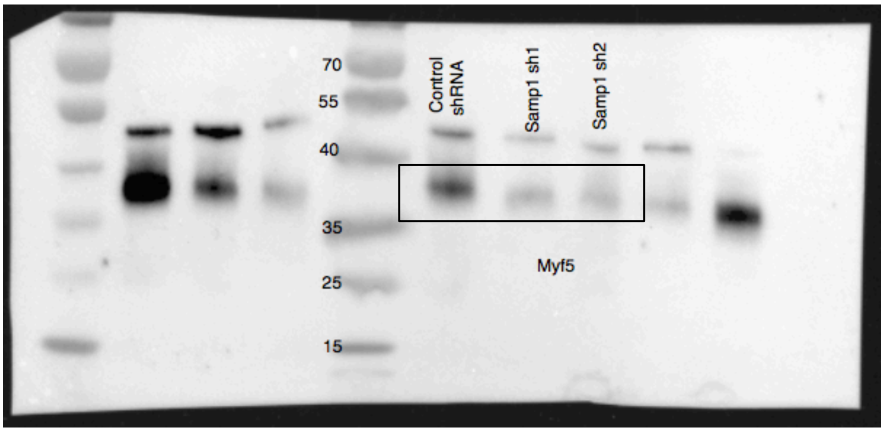

Figure 4A:

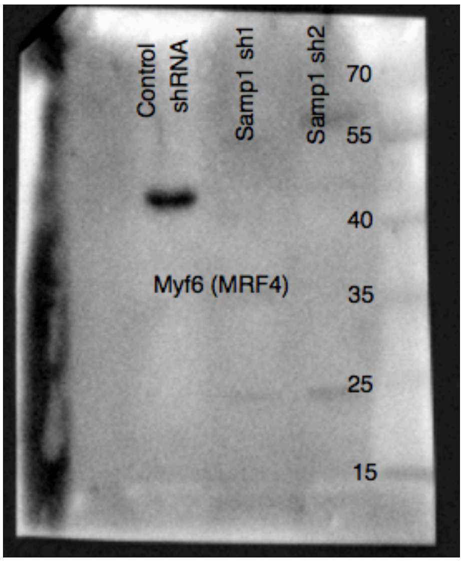

Figure 4A:

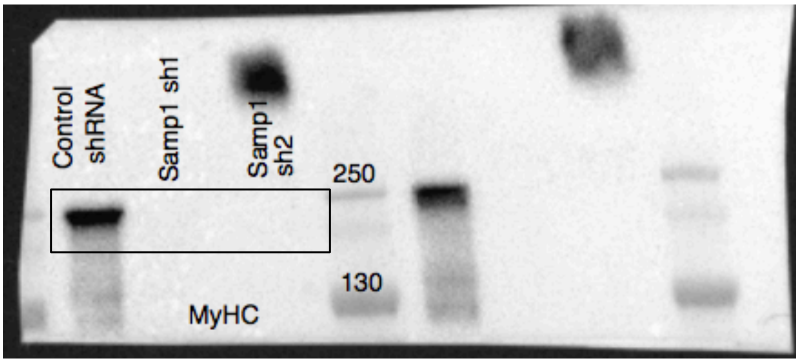

Figure 4A:

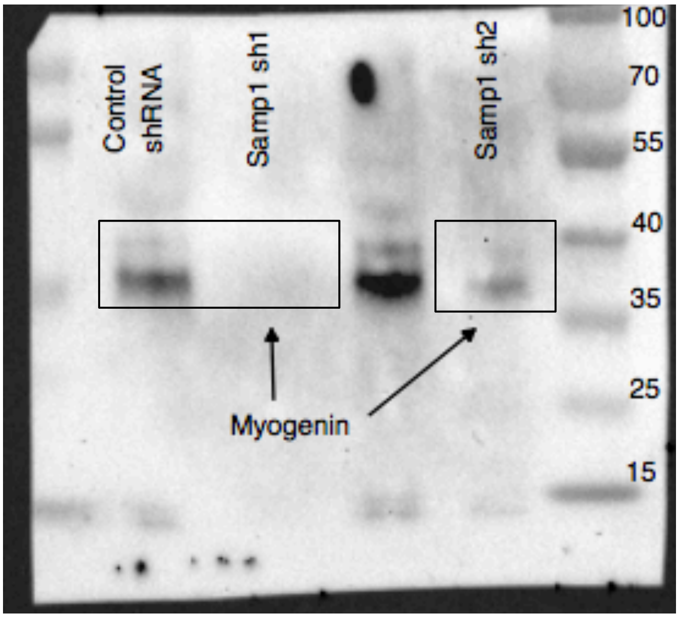

Figure 4A:

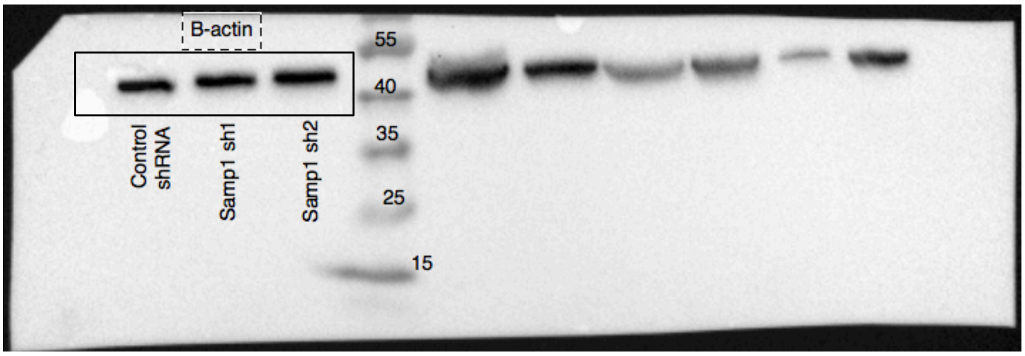

Figure 4C:

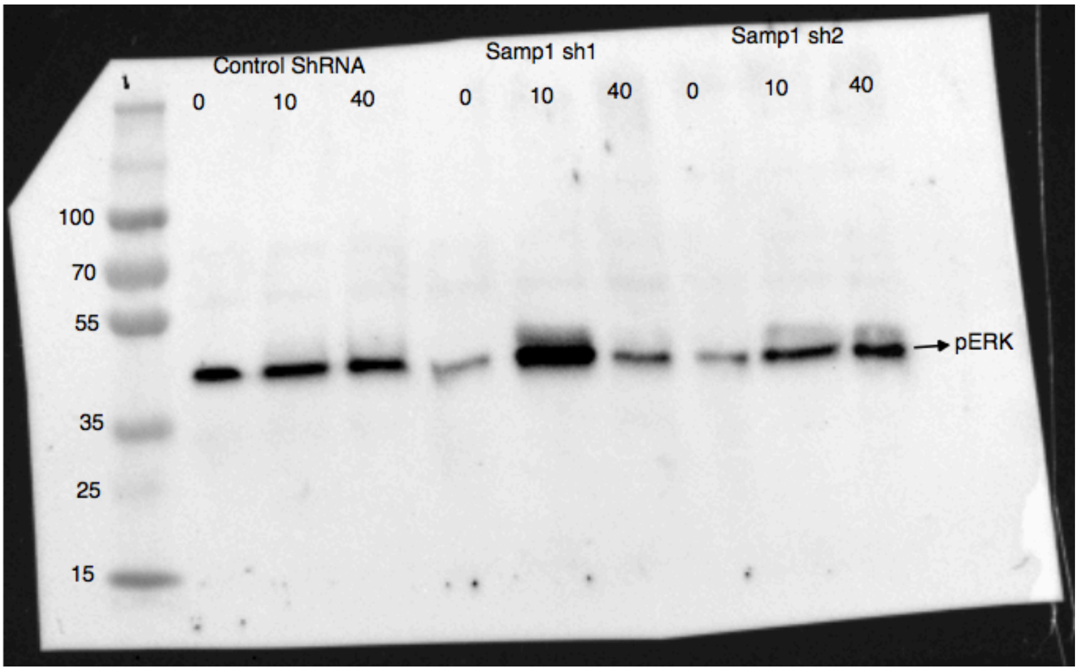

Figure 4C:

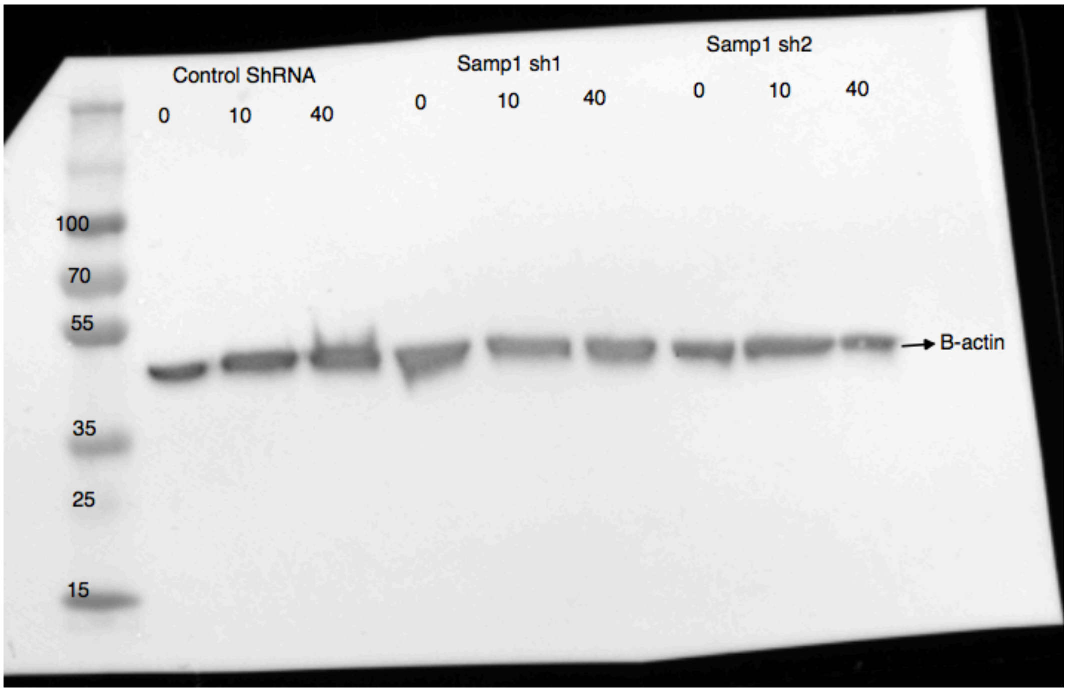

Figure S1A:

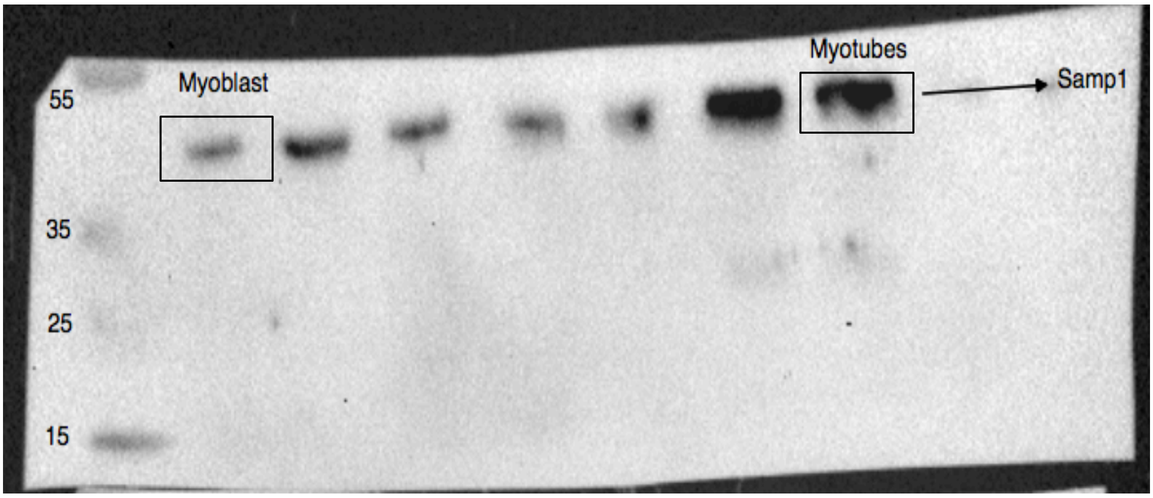

Figure S1A:

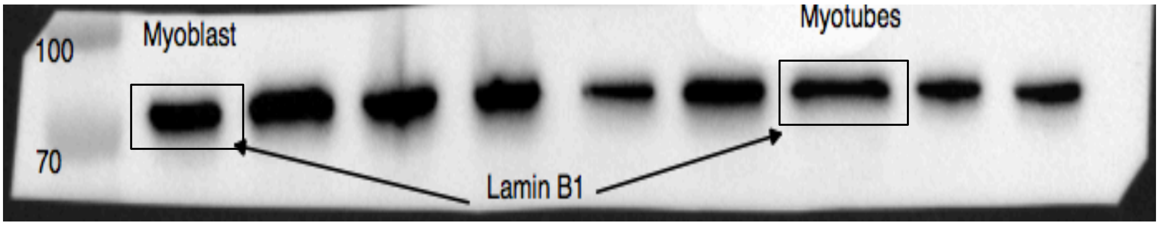

Figure S1A:

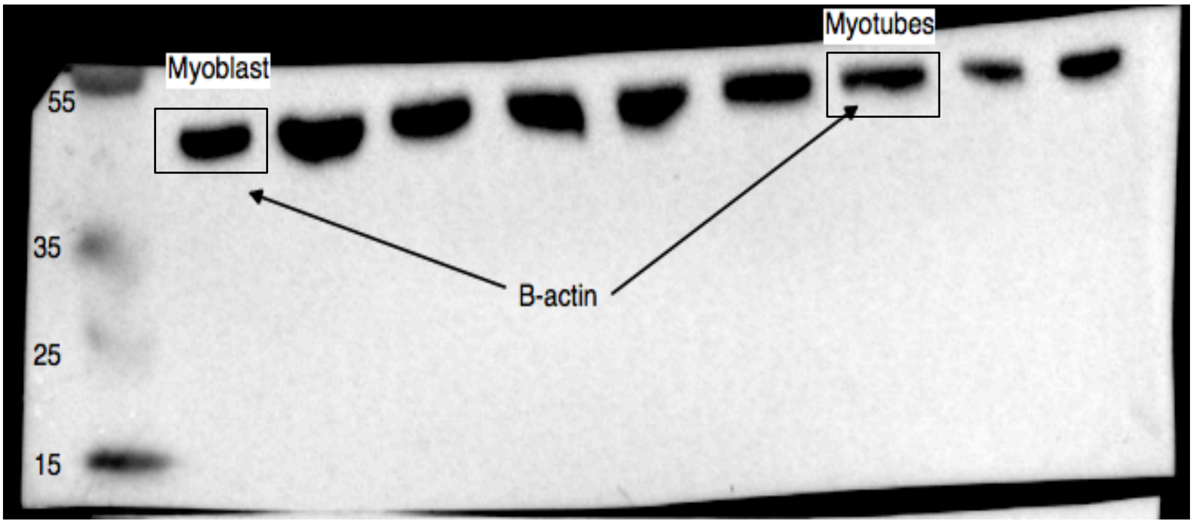

Supplementary Fig S3. **Uncropped Western blots shown in Figures 1B, 2A, 2D, 4A, 4C, and S1A.** Rectangles show the portion of each blots used for the respective figures.
